# Polycomb repressive complex 2 regulates skeletal growth by suppressing Wnt and TGF-β signalling

**DOI:** 10.1038/ncomms12047

**Published:** 2016-06-22

**Authors:** Fatemeh Mirzamohammadi, Garyfallia Papaioannou, Jennifer B. Inloes, Erinn B. Rankin, Huafeng Xie, Ernestina Schipani, Stuart H. Orkin, Tatsuya Kobayashi

**Affiliations:** 1 Endocrine Unit, Massachusetts General Hospital, Harvard Medical School, 50 Blossom Street, Boston, Massachusetts 02114, USA; 2 Division of Radiation and Cancer Biology, Department of Radiation Oncology, Center for Clinical Sciences Research, Stanford University, 875 Blake Wilbur Drive, Stanford, California, 94305, USA; 3 Department of Pediatric Oncology, Children's Hospital, Dana Farber Cancer Institute, 44 Binney Street, Boston, Massachusetts 02115, USA; 4 Department of Orthopaedic Surgery, Medical School, University of Michigan, Ann Arbor, 1500 E Medical Center Drive, Ann Arbor, Michigan 48109, USA

## Abstract

Polycomb repressive complex 2 (PRC2) controls maintenance and lineage determination of stem cells by suppressing genes that regulate cellular differentiation and tissue development. However, the role of PRC2 in lineage-committed somatic cells is mostly unknown. Here we show that Eed deficiency in chondrocytes causes severe kyphosis and a growth defect with decreased chondrocyte proliferation, accelerated hypertrophic differentiation and cell death with reduced *Hif1a* expression. Eed deficiency also causes induction of multiple signalling pathways in chondrocytes. Wnt signalling overactivation is responsible for the accelerated hypertrophic differentiation and kyphosis, whereas the overactivation of TGF-β signalling is responsible for the reduced proliferation and growth defect. Thus, our study demonstrates that PRC2 has an important regulatory role in lineage-committed tissue cells by suppressing overactivation of multiple signalling pathways.

## Abstract (web summary)

Eed is a polycomb repressive complex 2 component involved in stem cell lineage determination, but little is known about its role in lineage committed cells. Here the authors show that chondrocyte-specific Eed KO mice have skeletal growth defects related to induction of Wnt and TGF-β signalling.

## Graphical abstract

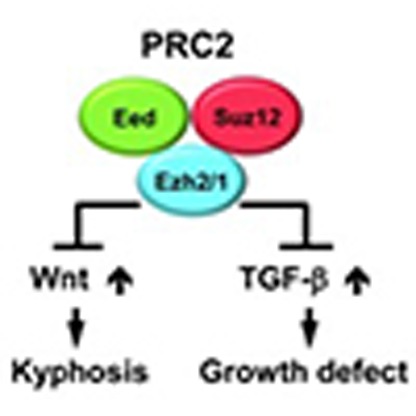

Most mineralized bone is formed through endochondral bone formation in which the growth plate, a cartilage template formed by chondrocytes, is continuously replaced by mineralized bone. Chondrocytes differentiate from multi-potent mesenchymal progenitors. After lineage commitment, growth plate chondrocytes go through multiple maturation steps to differentiate into post-mitotic hypertrophic chondrocytes^1^,^2^.

Proliferation and differentiation of growth plate chondrocytes is tightly controlled by multiple signalling systems^3^, including Indian hedgehog, parathyroid hormone-related peptide^1^,^4^, fibroblast growth factor^2^,^5^, C-type natriuretic peptide^6^, insulin-like growth factor^7^, bone morphogenetic protein, transforming growth factor-β (TGF-β)^8^,^9^ and Wnt signalling^10^,^11^. These extracellular signalling molecules are further mediated by specific and common intracellular signalling pathways, including the mitogen-activated protein kinase and phosphoinositide 3 kinase pathways.

Multiple epigenetic mechanisms regulate gene expression and thus control a variety of biological processes^12^,^13^,^14^. Altering chromatin structure via histone modification is a major epigenetic mechanism affected by polycomb group proteins. Polycomb repressive complex 2 (PRC2), whose core components include Ezh1/2 (enhancer of zeste homologue1/2), Eed (embryonic ectoderm development) and Suz12 (suppressor of zeste 12 homologue), catalyses tri-methylation of lysine 27 of histone 3 (H3K27me3), and silences chromatin^15^. These three components are essential for the methyltransferase activity of PRC2. PRC2 regulates expression of the key differentiation-related genes to control differentiation of embryonic stem cells (ESCs)^14^ and several tissue-specific stem/progenitor cells including haematopoietic stem cells^16^,^17^,^18^, neural stem cells^19^,^20^, muscle stem cells^21^ and epidermal stem cells^22^,^23^. These findings are in line with the notion that PRC2-mediated chromatin silencing controls cell fate transition of stem/progenitor cells^24^. However, the role of PRC2 in lineage-committed somatic cells is not clear.

Here we delete *Eed* in lineage-committed chondrocytes to investigate the role of PRC2 in skeletal development. We show that PRC2 has an essential role in regulating proliferation and differentiation of growth plate chondrocytes by suppressing multiple signalling pathways.

## Results

### *Eed* deletion in chondrocytes causes skeletal defects

**Figure 1:**
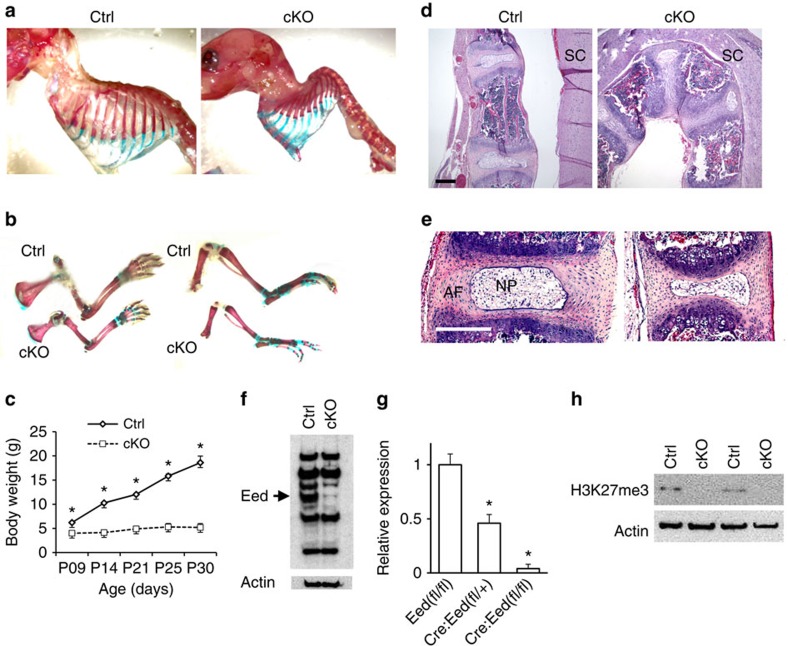
Conditional *Eed* deletion in chondrocytes causes skeletal defects. (**a**,**b**) Whole-mount skeletal preparation of littermate control (Ctrl) and *Eed* cKO mice at P11. Both male and female cKO mice show severe kyphosis (*Upper*) and shortening of long bones of forelimbs (*lower left*) and hind limbs (*lower right*). (**c**) cKO mice show impaired growth. A majority of cKO mice dies by P21. Male and female mice were combined. *n*=3–6; *P<0.05 versus Ctrl, error bars show the s.e.m., unpaired Student's *t*-test was used. (**d**) Sagittal sections of thoracic vertebrae of mice at P28.5. SC, spinal cord. (**e**) Intervertebral discs are also affected. *Eed* cKO mice show reduced size of the annulus fibrosus (AF) and nucleus pulposus (NP). (**f**) Immunoblot analysis using protein lysate of primary rib chondrocytes cultured overnight shows an efficient reduction in the Eed protein in cKO chondrocytes. The arrow indicates the signal specific to Eed. (**g**) The *Eed* RNA expression level determined by qPCR in chondrocytes from indicated genotypes. (**h**) H3K27me3 is absent in primary rib chondrocytes of cKO mice (Supplementary Fig. 6; the uncropped blot result images). Scale bars, 200 μm.

In order to investigate the role of PRC2 in skeletal development, we ablated the *Eed* gene in chondrocytes using floxed *Eed* mice and *Col2-Cre* transgenic mice in which Cre recombinase is expressed under the control of a mouse *Col2a1* promoter. *Eed* conditional knockout (*Col2-Cre:Eedfl/fl*, cKO) mice were born and survived postnatally, but showed growth impairment, shortening of long bones and severe kyphosis, and usually died by 4 weeks (Fig. 1a–c). The mutant thoracic spine was severely deformed (Fig. 1d). In addition to vertebral bodies, intervertebral discs that are also formed by cells derived from *Col2a1*-positive progenitors showed a reduction in size in *Eed* cKO mice (Fig. 1e). Using primary chondrocytes isolated from ribs, we confirmed efficient elimination of Eed at the protein and RNA levels and H3K27me3 in *Eed* cKO chondrocytes (Fig. 1f–h).

### PRC2 regulates chondrocyte proliferation and differentiation

**Figure 2:**
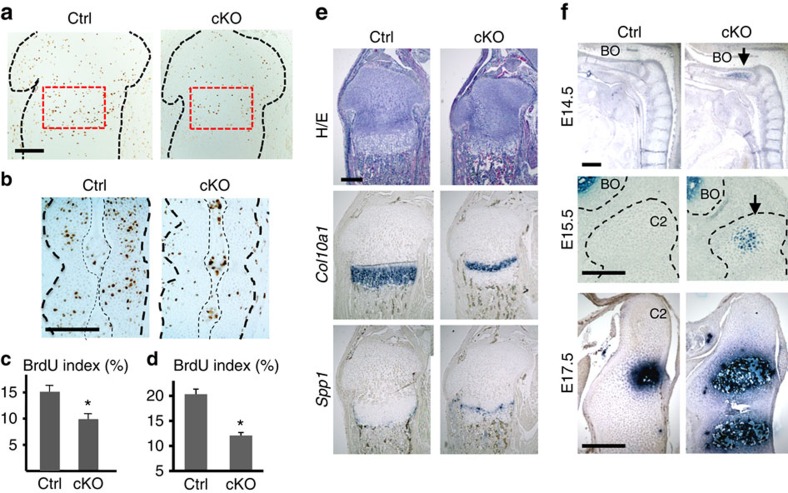
Proliferation and differentiation defects in *Eed*-deficient chondrocytes. (**a**,**c**) BrdU incorporation is significantly reduced in growth plate chondrocytes of *Eed* cKO mice. Black dotted lines indicate the contour of the proximal epiphysis of tibiae. The BrdU labelling index in the proliferating zone, indicated by red-dotted lines, was counted in P 1.5-old littermate control and cKO mice. (**b**,**d**) BrdU incorporation is significantly reduced in the cartilage anlage of the cKO spinal primordium at E15.5. Thick black dotted lines indicate the contour of the spinal primordium at the level of C4 and C5. Thin dotted lines indicate the notochord. *n*=3; *P<0.05 versus Ctrl. (**e**) The *Col10a1*-expressing hypertrophic chondrocyte domain is reduced in the P1.5-old cKO tibial growth plate. An unresorbed hypertrophic chondrocyte layer beneath the *Spp1* domain is occasionally present in cKO mice. (**f**) Sagittal sections of the spine show premature differentiation of *Col10a*-expressing hypertrophic chondrocytes (arrows) in cKO vertebral bones at indicated ages. BO, basi-occipital bone; C2, second cervical vertebra (axis). Scale bars, 200 μm.

While the overall structure of growth plates is relatively well preserved, *Eed* cKO chondrocytes of tibial growth plates and fetal vertebrae showed significant decreases in cell proliferation (Fig. 2a–d). This proliferation defect likely led to a reduction in the number of type X collagen (*Col10a1*)-expressing hypertrophic chondrocytes that are differentiated from proliferating chondrocytes (Fig. 2e). The marker of terminally differentiated hypertrophic chondrocytes, *Spp1*, was found beneath the hypertrophic layer both in control and *Eed* cKO growth plate; however, we occasionally observed chondrocytes below the *Spp1* domain, suggesting delayed cartilage resorption (Fig. 2e). In order to investigate the effect of *Eed* deficiency on chondrocyte differentiation, we examined the initial appearance of hypertrophic chondrocytes in the embryonic axial skeleton. Appearance of *Col10a1*-expressing hypertrophic chondrocytes was advanced in *Eed* cKO mice at multiple developmental stages (Fig. 2f), demonstrating that *Eed* deficiency accelerates hypertrophic differentiation.

### *Eed* deletion decreases *Hif1a* and induces cell death

**Figure 3:**
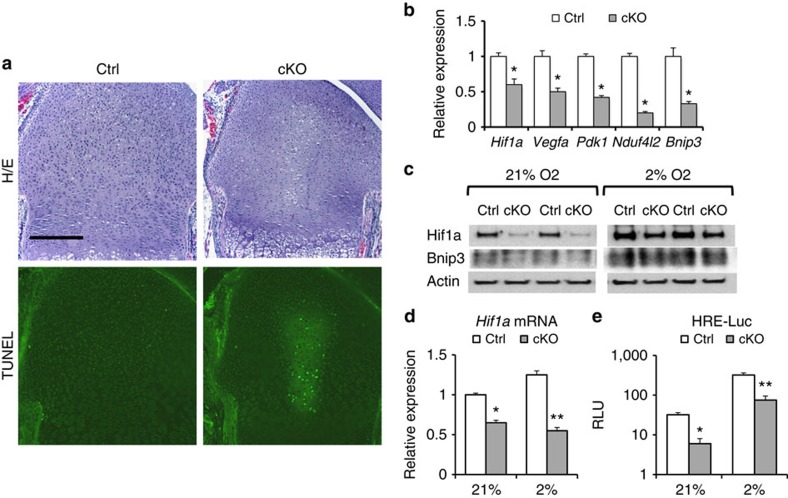
*Eed* deletion impairs hypoxic adaptation of chondrocytes. (**a**) Haematoxylin/eosin (*top*) and TdT-mediated dUTP nick end labeling (TUNEL; *bottom*)-stained sections of the distal femoral growth pate of P1.5-old mice. Cell death is detected in the central area of the *Eed* cKO growth plate. (**b**) Expression levels of *Hif1a* and indicated Hif1a target genes were reduced in primary *Eed* cKO chondrocytes cultured in the normoxic condition. RNA expression levels were determined by qRT–PCR. Expression levels were normalized to *Actb* levels. *n*=4; * *P*<0.05 versus Ctrl. (**c**) Expression of Hif1a and Bnip3 is reduced in primary cKO rib chondrocytes both in normoxic (21% O_2_) and hypoxic (2% O_2_) conditions. (**d**) The *Hif1a* mRNA level is significantly reduced in cKO chondrocytes. *Hif1a* levels were normalized by 18S ribosomal RNA. *n*=4; *P<0.05, **P<0.01 versus Ctrl. (**e**) Hif1a activity, assessed by a Hif1a-responsive luciferase reporter (HRE-luc), was reduced in cKO in both normoxic and hypoxic conditions. *n*=6; *P<0.05, **P<0.01 versus Ctrl. Scale bars, 200 μm. Error bars show the s.e.m., unpaired Student's *t*-test was used.

We also found that *Eed* cKO mice often showed reduced cellular density in the central area of epiphyseal growth plates at early postnatal stages (Fig. 3a). TdT-mediated dUTP nick end labelling staining demonstrated cell death in this region. The growth plate is a hypoxic tissue where most of the hypoxic chondrocytes are located in the centre of the columnar proliferating chondrocyte layer. The functional activity of the hypoxia-inducible transcription factor 1α (Hif1a) is required to maintain chondrocyte viability within the central region of the growth plate^25^. On the basis of the similarity in the pattern of cell death between *Eed*-deficient and *Hif1a*-deficient growth plates, we examined expression of Hif1a and the Hif1a downstream target genes. RNA expression of Hif1a and its known target genes were downregulated in Eed cKO chondrocytes at the normoxic condition (Fig. 3b). Protein expression levels of Hif1a and the Hif1a target, Bnip3, were decreased in cKO primary chondrocytes both in the normoxic (21% oxygen) and hypoxic (2% oxygen) conditions (Fig. 3c). Hypoxia-dependent Hif1a protein stabilization, a major regulatory mechanism of Hif1a expression, was intact in *Eed* cKO chondrocytes, since hypoxia efficiently increased the Hif1a protein level and Hif1a activity in both control and cKO chondrocytes (Fig. 3c,e). Thus, the reduction in Hif1a expression in *Eed*-deficient chondrocytes appeared to be mainly caused by a decrease in *Hif1a* mRNA. These findings suggest that transcriptional regulation of Hif1a by PRC2 is necessary for chondrocyte viability within the central region of the growth plate.

### PRC2 controls activities of multiple signalling pathways

To understand the molecular mechanism, first we performed chromatin immunoprecipitation (ChIP) followed by sequencing (ChIP-seq) to identify genes that carried the H3K27me3 mark in chondrocytes (Gene Expression Omnibus (GEO) #GSE67132 and #GSE76467; Supplementary Data 1). We found that 3,925 genes were associated with H3K27me3 in chondrocytes (Supplementary Fig.1a and Supplementary Data 1). As found in ESCs^26^, genes encoding transcription factors and signalling molecules were preferred PRC2 targets in chondrocytes as well. We also performed gene expression analysis to identify genes whose expression was altered upon *Eed* deletion (GEO #GSE66862). We found that 1,817 annotated genes were upregulated more than 1.25-fold in *Eed*-null chondrocytes (Supplementary Fig. 1 and Supplementary Data 2). Among these, 433 genes carried the H3K27me3 mark, suggesting that the majority of deregulated genes in *Eed*-deficient chondrocytes were indirectly regulated by PRC2 (Supplementary Data 1). The finding that only 433 among 3,925 genes marked with H3K27me3 were derepressed in *Eed*-deficient chondrocytes also suggests that a majority of PRC2 target genes are repressed by other mechanisms.

**Figure 4:**
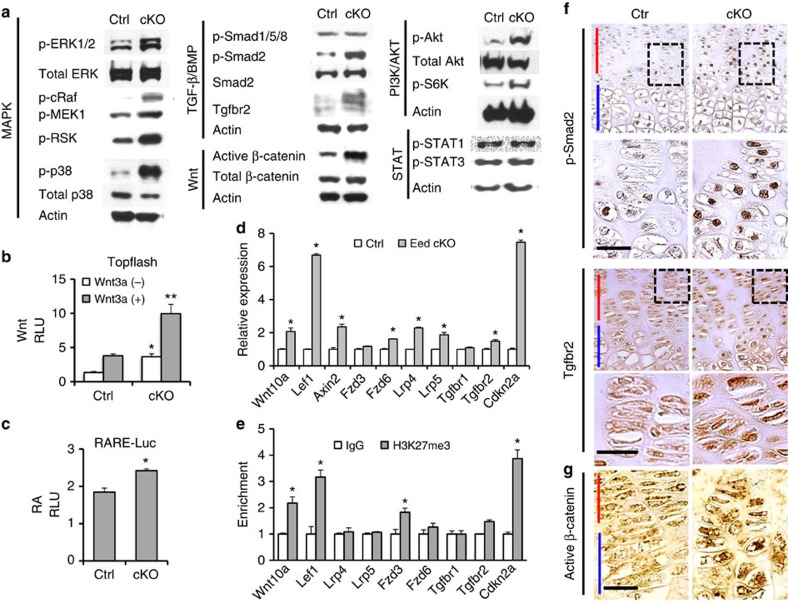
*Eed* deletion causes aberrant activation of multiple signalling pathways. (**a**) The basal levels of major signalling pathways in primary rib chondrocytes were determined by immunoblot analysis using indicated phospho-specific antibodies. (**b**) Topflash Wnt reporter assay shows upregulation of Wnt activity in cKO chondrocytes both at the basal and Wnt3a-stimulated conditions. After transfection, cells were cultured with or without Wnt3a (20 ng ml^−1^) for 24 h. *n*=6; *P<0.05, **P<0.01 versus Ctrl. (**c**) RA-responsive luciferase assay (RARE-Luc) shows modest upregulation in RA signalling in cKO chondrocytes. *n*=6; *P<0.05 versus Ctrl. (**d**) RNA expression of indicated genes in primary rib chondrocytes were determined by qRT–PCR. *n*=4; *P<0.05 versus Ctrl. (**e**) H3K27me3 ChIP-PCR analysis of indicated genes. *n*=4; *P, 0.05 versus IgG control. (**f**) Immunostaining for p-Smad2 and Tgfbr2 on tibial growth plate sections of Ctrl and cKO mice. Representative pictures of three independent experiments are shown. (**g**) Immunostaining for non-phosphorylated β-catenin (active form) on tibial growth plate sections from Ctrl and cKO. Representative pictures of three independent experiments are shown. Red bar indicates the proliferating chondrocyte zone and blue bar indicates the hypertrophic chondrocyte zone of the growth plate. Scale bars, 20 μm. Error bars show the s.e.m., unpaired Student's *t*-test was used.

Since genes encoding signalling molecules account for a considerable fraction of H3K27me3-marked genes in chondrocytes, we determined the basal signalling status of major signalling pathways in *Eed*-deficient chondrocytes. We found that *Eed* deficiency caused overactivation in multiple signalling pathways (Fig. 4a–c and Supplementary Fig. 2b). Phosphorylation of signalling molecules of the extracellular-signal-regulated kinase (ERK), p38 mitogen-activated protein kinase, phosphoinositide 3 kinase and TGF-β pathways was upregulated. Increases in active β-catenin and Wnt reporter activity indicated the upregulation of canonical Wnt signalling in *Eed*-deficient chondrocytes (Fig. 4a,b,g). We also found a modest upregulation in retinoic acid (RA) signalling assessed by a luciferase reporter assay. As found in the microarray analysis (Supplementary Data 2), we found upregulation in *Wnt10a* and *Lef1*, of which genomic loci were associated with H3K27me3 (Fig. 4e and Supplementary Fig. 1b), and the Wnt target gene, *Axin2* (Fig. 4d). We also found upregulation of Wnt receptors, including Fzd6 and Lrp5 (Fig. 4d); however, these genes did not carry the H3K27me3 mark, suggesting that PRC2 loss indirectly increased expression of these genes. With regard to the TGF-β signalling pathway, we found that Tgfbr2 was upregulated at both mRNA and protein levels (Fig. 4a,d,f). We did not find significant H3K27me3 modification at the Tgfbr2 gene locus; thus, Tgfbr2 upregulation in chondrocytes is likely an indirect consequence of the loss of PRC2 function.

### TGF-β suppression rescues proliferation and growth defects

**Figure 5:**
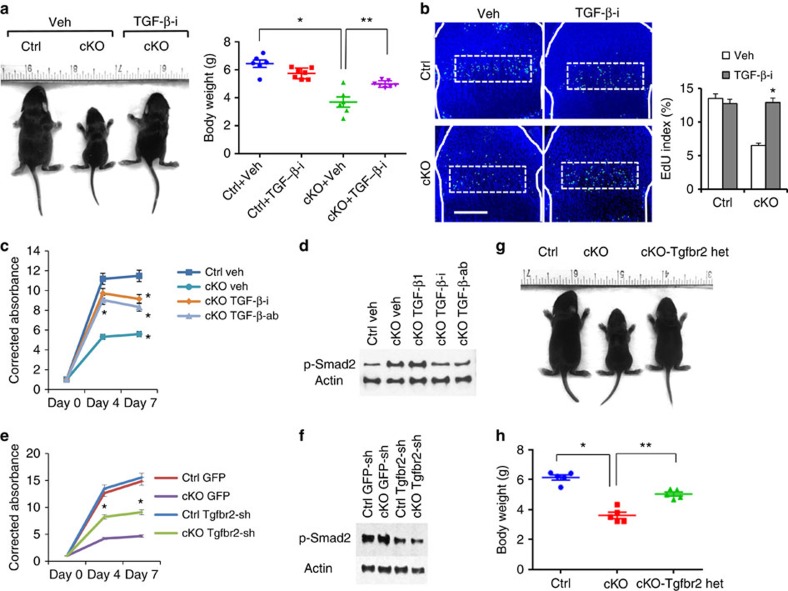
TGF-β inhibition rescues the growth defect of *Eed* cKO mice. (**a**) The TGF-β receptor inhibitor, Ly364947, was injected daily into pregnant or nursing mothers from E14.5 through P7.5. Mice at P7.5 are shown. Body weight was measured at P7.5. *n≥*6; *P<0.05 versus Ctrl, **P<0.05 versus vehicle-treated cKO. (**b**) EdU staining of proximal tibial growth plates. The fraction of EdU-positive nuclei was calculated in proliferating columnar chondrocyte zone (dotted lines). White solid lines indicate the epiphyseal bone contour. *n*=3; *P<0.05 versus vehicle-treated cKO mice. Scale bars, 200 μm. (**c**) Cell proliferation assay on primary chondrocytes isolated from wild-type (Ctrl) and *Eed* cKO (cKO) mice, treated with dimethylsulphoxide (DMSO; Veh) or a TGF-β receptor inhibitor (TGF-β-i; Ly364947, 0.2 μM), TGF-β ligand-neutralizing antibody (TGF-β-ab; 5 μg ml^−1^) or TGF-β1 (20 ng ml^−1^). Treatment with Ly364947 and TGF-β-ab significantly ameliorated the proliferation defect of cKO cells. *n*=3; *P<0.01 and **P<0.05. (**d**) Treatment with TGF-β-i and TGF-β-ab decreases the p-Smad2 level. (**e**) Primary rib chondrocytes from Ctrl and cKO mice were infected with retroviruses expressing *Tgfbr2* shRNA (*Tgfbr2*-sh) or eGFP (enhanced green fluorescent protein; GFP). Tgfbr2 knockdown using Tgfbr2-sh2 virus in cKO chondrocytes significantly increases proliferation. *n*=6; *P<0.05. (**f**) The p-Smad level is decreased in *Tgfbr2* knockdown chondrocytes. (**g**) *Tgfbr2* heterozygosity (cKO-*Tgfbr2* het) significantly improves animal growth of *Eed* cKO mice. (**h**) Body weight of control (Ctrl), *Eed* cKO (cKO) and *Eed* cKO mice missing one allele of *Tgfbr2* (cKO-*Tgfbr2* het) was measured at P7.5. Mice with conditional *Tgfbr2* heterozygous deletion alone (*Col2*_**-_*Cre:Tgfbr*^*loxP/+*^) show no noticeable skeletal abnormalities, as also previously described^37^. *n*≥5; *P<0.01 and **P<0.05. Error bars show the s.e.m., unpaired Student's *t*-test was used.

Because the ERK, p38 and TGF-β pathways, which were upregulated in *Eed* cKO chondrocytes, were shown to regulate endochondral bone growth^27^,^28^,^29^,^30^, we inhibited these signalling pathways *in vivo* to test whether upregulation of these signalling pathways contributed to the skeletal abnormalities of *Eed* cKO mice. Whereas inhibition of the ERK or p38 signalling pathway had no effects on the skeletal defects of *Eed* cKO mice (Supplementary Fig. 2c), inhibition of TGF-β signalling using the TGF-β receptor inhibitor, Ly364947, ameliorated the growth defect during early postnatal stages (Fig. 5a). Analysis of the growth plate of *Eed* cKO mice treated with Ly364947 revealed a decrease in phospho-Smad2 immunostaining and a significant increase in chondrocyte proliferation compared with vehicle-treated *Eed* cKO mice (Fig. 5b and Supplementary Fig. 4d). We also assessed the effect of TGF-β signalling suppression on chondrocyte proliferation *in vitro*. TGF-β signalling inhibition using TGF-β receptor inhibitor (Ly364947) or neutralizing antibody against TGF-β ligands (1D11) significantly ameliorated proliferation defect of *Eed* cKO chondrocytes *in vitro* (Fig. 5c,d). Because *Tgfbr2* was significantly upregulated in *Eed* cKO chondrocytes, to test whether upregulation of *Tgfbr2* was responsible for the proliferation defect of *Eed* cKO chondrocytes, we knocked down *Tgfbr2* using retroviruses expressing small hairpin RNAs (shRNAs) in primary rib chondrocytes *in vitro*. With ∼60% infection efficiency, *Tgfbr2* expression was reduced by 40–60% (Supplementary Fig. 5a). *Tgfbr2* knockdown in *Eed* cKO chondrocytes showed a significant increase in proliferation *in vitro* (Fig. 5e,f). We also found that the well-known PRC2 target, *Cdkn2a* (*Ink4a/Arf*), encoding negative cell cycle regulators^31^ was upregulated in *Eed* cKO chondrocytes (Fig. 4d,e). To evaluate the role of *Cdkn2a* upregulation in chondrocyte proliferation, we knocked down *Cdkn2a* in *Eed* cKO chondrocytes; *Cdkn2a* knockdown had little effect in chondrocytes unlike *Tgfbr2* knockdown despite the similar knockdown efficiencies (Supplementary Fig. 5b,c).

To further investigate the role of the *Tgfbr2* upregulation in *Eed* cKO mice *in vivo*, we generated compound conditional mutant mice missing Eed and one allele of *Tgfbr2*. *Tgfbr2* heterozygosity significantly improved cellular proliferation and animal growth in *Eed* cKO mice (Fig. 5g,h and Supplementary Fig. 5d). These results demonstrate that the overactivation of TGF-β signalling because of the upregulation of *Tgfbr2* plays a causal role for the proliferation defect of growth plate chondrocytes and growth impairment of *Eed* cKO mice.

### Wnt signalling overactivation causes kyphosis

**Figure 6:**
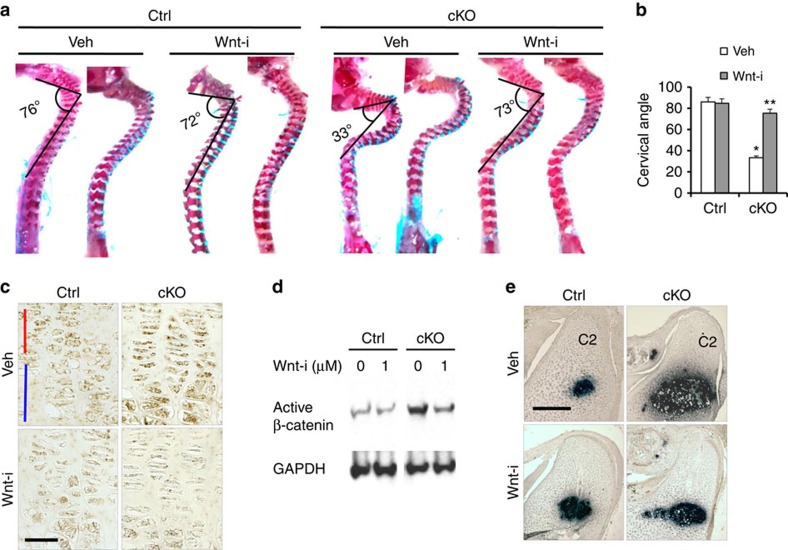
Wnt inhibition rescues kyphosis of *Eed* cKO mice. (**a**) The effect of Wnt inhibition on kyphosis was assessed by the spinal cervical angle in P7.5-old mice. C59 Wnt inhibitor (Wnt-i) treatment from E14.5 through P7.5 significantly ameliorated kyphosis in *Eed* cKO mice. (**b**) Cervical angle measurements. *P<0.05 versus vehicle-treated Ctrl. **P<0.05 versus vehicle-treated cKO. Error bars show the s.e.m., unpaired Student's *t*-test was used. (**c**) Expression of active (non-phosphorylated) β-catenin of growth plate sections of Ctrl and cKO mice was assessed 6 h after C59 (Wnt-i) or vehicle injection. A significant reduction of active β-catenin protein was observed in C59-treated animals. Scale bar, 20 μm; red bar indicates the proliferating chondrocyte zone and blue bar indicates the hypertrophic zone. (**d**) *In vitro* treatment with C59 (Wnt-i) decreased the active (non-phosphorylated) β-catenin level in *Eed* cKO primary rib chondrocytes. Chondrocytes were cultured for 24 h in the presence of C59 (Wnt-i). (**e**) *Col10a1 in situ* hybridization on sagittal sections of the cervical spine of E17.5 embryos shows suppression of accelerated hypertrophic differentiation. C59 was injected into pregnant mothers from E 14.5 through E 17.5. C2, second cervical vertebra. Scale bar, 200 μm.

Although TGF-β inhibition rescued the growth defect, inhibition of the TGF-β, ERK or p38 signalling pathways did not improve the spinal deformity (Supplementary Fig. 2c). Because the Wnt pathway, which was also upregulated in *Eed* cKO chondrocytes, was shown to regulate cartilage development by controlling chondrocyte differentiation^11^,^29^,^30^, we inhibited Wnt signalling using the porcupine inhibitor, C59. Daily treatment of C59 during fetal and neonatal stages significantly ameliorated the spinal deformity (Fig. 6a,b). Analysis of developing vertebrae of *Eed* cKO mice treated with C59 revealed significant suppression of premature hypertrophic differentiation in cKO mice (Fig. 6e). C59 treatment also rescued the premature closure of the growth plate between the vertebral body and transverse processes in the *Eed* cKO spine (Supplementary Fig. 3a). We confirmed that C59 treatment efficiently suppressed canonical Wnt signalling in *Eed*-deficient chondrocytes *in vivo* and *in vitro* (Fig. 6c,d). In long bones, although Wnt inhibitor treatment rescued premature hypertrophic differentiation in the secondary ossification centre of the *Eed*-deficient tibial epiphysis (Supplementary Fig. 3b), it did not rescue the growth defect (Supplementary Fig. 3c) or the cell proliferation defect (Supplementary Fig. 3d).

## Discussion

The genome-wide mapping of PRC2-binding sites and H3K27 tri-methylation in ESCs revealed that PRC2 regulate numerous genes encoding the key developmental regulators, demonstrating the critical role of PRC2 in developmental and cellular differentiation processes^32^,^33^,^34^. While the role of PRC2 in embryonic and tissue-specific stem/progenitor cells has been extensively studied^14^, its role in lineage-committed, differentiated somatic cells is largely unexplored.

The role of PRC2 in mesenchymal stem/progenitor cells of the skeletal system has been investigated in multiple models. *Ezh2* deficiency in the early limb mesenchymal stem/progenitor cells reduces their proliferation, increases cell death and alters anteroposterior specification presumably because of the deregulation of patterning-regulating genes, such as *Hox* genes^35^. Likewise, *Ezh2* deletion in neural crest cells causes derepression of *Hox* genes, impairs differentiation of osteochondro progenitors and results in craniofacial defects^36^. However, specific roles of PRC2 in lineage-committed, skeletal cells are not demonstrated by these studies. In this study we show that PRC2 continuously plays an important role in regulation of cellular function of chondrocytes after lineage commitment by suppressing multiple signalling pathways.

Major skeletal phenotypes of *Eed* cKO mice include a growth defect and kyphosis. The observation that Wnt inhibitor treatment significantly ameliorated the accelerated chondrocyte differentiation and spinal deformity of *Eed* cKO mice strongly suggests that premature differentiation into post-mitotic hypertrophic differentiation plays a causal role for kyphosis. The acceleration of hypertrophic differentiation likely reduces the net number of chondrocytes, induces premature ossification and thus compromises spinal development.

In contrast to Wnt inhibition, while TGF-β inhibitor treatment did not rescue the spinal deformity, it did rescue the proliferation defect and the growth defect in *Eed* cKO mice; thus, suppression of TGF-β signalling by PRC2 is essential for normal chondrocyte proliferation and animal growth. This finding is in line with previous studies in which TGF-β treatment decreases and its inhibition increases chondrocyte proliferation *in vivo*^27^,^28^. It is worth pointing out that the effect of TGF-β signalling on chondrocyte proliferation is likely bitropic, as Tgfbr2 conditional deletion can also reduce chondrocyte proliferation possibly depending on the differentiation status^37^,^38^. We also found that treatment of the TGF-β inhibitor at higher concentrations decreased chondrocyte proliferation as opposed to lower doses, suggesting a dose-dependent effect of TGF-β signalling on chondrocyte proliferation (Supplementary Fig. 5e). PRC2 is known to regulate cell proliferation by suppressing cell cycle inhibitors, such as *Cdkn2a* (*Ink4a/*Arf), and^16^,^18^ *Cdkn2a* deletion partially rescues phenotypes caused by PRC2 deficiency^16^,^18^. We indeed found that *Cdkn2a* was upregulated in *Eed* cKO chondrocytes. Although we were not able to rescue the proliferation defect of *Eed* cKO chondrocytes by shRNA-mediated *Cdkn2a* knockdown *in vitro*, it is still possible that upregulation of cell cycle inhibitors contributes to the proliferation defect of *Eed* cKO chondrocytes *in vivo*. Nevertheless, our data demonstrate the central role of the TGF-β upregulation in the proliferation defect of *Eed* cKO chondrocytes.

Another unique phenotype of chondrocyte-specific *Eed* cKO mice is the cell death in the central region of the growth plate. The central region of the growth plate is hypoxic, and hypoxia adaptation via Hif1a is essential for chondrocyte survival in this area^25^. The Hif1a level is mainly controlled at the post-transcriptional level by oxygen-dependent protein^39^. Relatively little is known about transcriptional regulation of *Hif1a* expression except that a few signalling pathways, such as RA^40^, NF-κB^41^ and calcineurin/NFATc pathways^42^, were reported to regulate *Hif1a* transcription. We tested the effects of pathway-specific inhibitors on *Hif1a* expression in control and *Eed* cKO primary chondrocytes (Supplementary Fig. 4a). We were not able to restore expression of *Hif1a* or Hif1a target genes by inhibiting signalling pathways that were upregulated in *Eed*-deficient chondrocytes. Thus, the mechanism by which *Eed* deficiency decreases *Hif1a* transcripts is not clear at the moment. An association between chondrocyte death and TGF-β signalling was also reported in mice missing Smad7, a negative regulator of TGF-β and bone morphogenetic protein signaling, in growth plates^43^. However, Smad7 deficiency showed an increase in Hif1a protein expression, and therefore the cell death in the Smad7-null growth plate is likely caused by the Hif1a-independent mechanism. We found that TGF-β inhibition *in vivo* decreased the occurrence of cell death in *Eed* cKO mice (Supplementary Fig. 5); thus, it is possible that cell death in the *Eed* cKO growth plate is caused by a Hif1a-independent, TGF-β signalling-dependent pathway.

We demonstrate that upregulation of *Tgfbr2* expression is, at least in part, responsible for growth and proliferation defects of *Eed* cKO mice. This finding is in line with a recent study showing that PRC2 targets and suppresses *Tgfbr2* to facilitate mesenchymal–epithelial transition during reprogramming of fibroblasts into pluripotent stem cells^44^. However, we found that the *Tgfbr2* gene was not strongly marked with H3K27me3 (Supplementary Data 1); thus, its upregulation is likely indirectly caused by *Eed* deficiency in chondrocytes.

Wnt signalling plays an important role during endochondral development. Ablation of β-catenin (Ctnnb1), a critical mediator of the canonical Wnt signalling pathway, in growth plate chondrocytes delays hypertrophic differentiation^45^, whereas overexpression of a stable form of Ctnnb1 in chondrocytes accelerates it^11^. In this study, we found that inhibition of Wnt signalling rescued the acceleration of hypertrophic differentiation and kyphosis in *Eed* cKO mice, demonstrating the critical role of PRC2-mediated suppression of Wnt signalling in regulation of chondrocyte differentiation and normal skeletal development.

**Figure 7:**
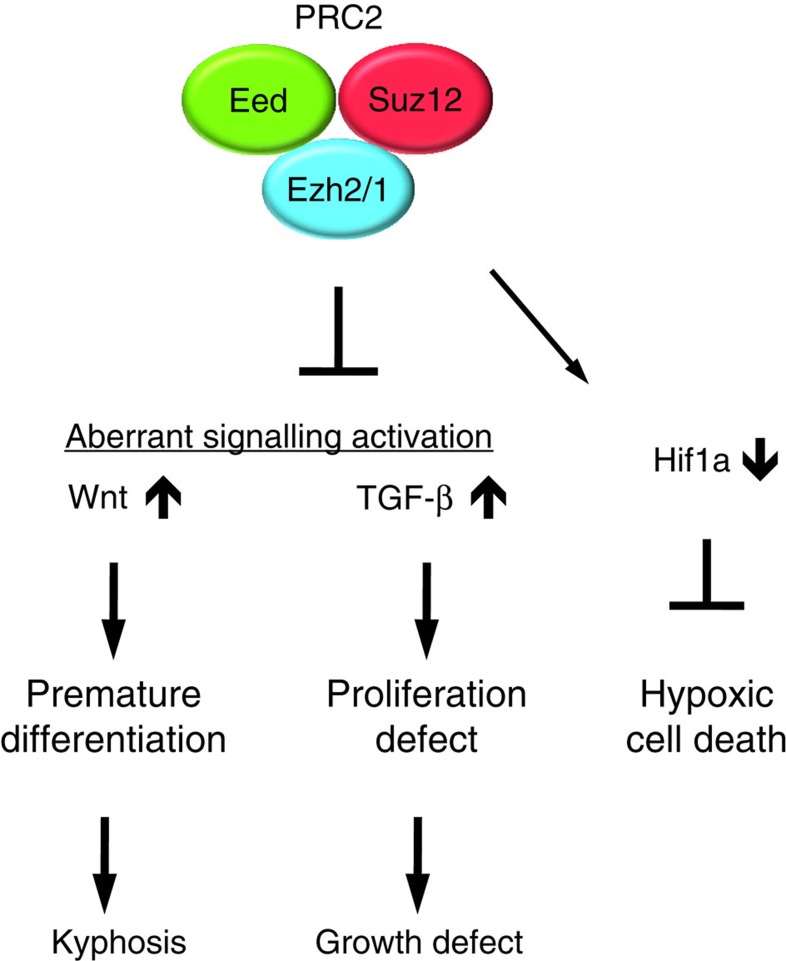
Signalling regulation by PRC2 in chondrocytes. PRC2 is necessary for normal skeletal growth. Derepression of PRC2 target genes causes aberrant overactivation of multiple signalling systems and reduces *Hif1a* expression. Overactivation of Wnt and TGF-β signalling pathways is primarily responsible for the skeletal abnormalities of mice with *Eed*-deficient chondrocytes.

In summary, this work demonstrates that the PRC2 continuously plays an important regulatory role in differentiation and proliferation of lineage-committed growth plate chondrocytes by suppressing Wnt and TGF-β signalling pathways (Fig. 7).

## Methods

### Mice

*Col2-Cre* transgenic mice^46^, floxed *Eed* mice^18^ and floxed *Tgfbr2* mice^47^ were previously described. Mice were in a mixed genetic background. Comparison between control and cKO mice was always made between littermates.

The MEK1 inhibitor, U0126, the p38 inhibitor, SB203580, the TGF-β receptor inhibitor, LY364947, and the Wnt inhibitors, C59 and XAV-939, were purchased from Selleckchem. The RA inhibitors, BMS493 and AGN193109, were purchased from Santa Cruz Biotechnology Inc. The neutralizing antibody against TGF-β ligands, 1D11, was purchased from R&D Systems. Inhibitors were first dissolved in dimethylsulphoxide according to the manufacturer's instructions, then diluted into 100 μl PBS and then injected daily into pregnant as well as nursing mothers intraperitoneally from E 14.5 through P9.5.

The animal experiments were approved by the Institutional Animal Care and Use Committee of the Massachusetts General Hospital and performed in accordance with the regulations and guidelines.

### Skeletal preparation and histological analysis

Skeletal preparation^48^, histological staining of paraffin-processed samples^49^ and *in situ* hybridization^50^ were performed using standard procedures. Kyphosis was assessed as previously described^51^; two lines were drawn from the vertebral body of the first thoracic vertebra (Th1) to the spinous process of the second cervical vertebra (C2) and that of Th12; the angle formed by these two lines were measured.

### Proliferation and cell death assays

For bromodeoxyuridine (BrdU) or ethynyldeoxyuridine (EdU) labelling, 50 μg g^−1^ body weight of BrdU or 20 μg g^−1^ of EdU was injected into mice intraperitoneally 2 h before killing. BrdU or EdU was detected using the BrdU *In Situ* Staining Kit or Click-iT EdU Alexa Fluor 488 Imaging Kit (Life Technologies). The BrdU or EdU labelling index was calculated as the ratio of BrdU- or EdU-positive nuclei over total nuclei in columnar proliferating chondrocytes of the growth plate. Cell death was evaluated on sections using the *In Situ* Cell Death Detection Kit (Roche) according to the manufacturer's instruction. *In vitro* cell proliferation assay was performed using the PrestoBlue cell viability reagent (Invitrogen).

### Primary chondrocyte isolation and culture

Primary rib chondrocytes were isolated from neonatal mice by collagenase digestion as previously described^52^. After overnight culture in DMEM containing 10% fetal calf serum, cells were subjected to downstream analysis.

### Luciferase reporter assay

For luciferase assay, cells were trypsinized, replated in 96-well culture dishes and transfected with the 0.2 μg of Hif1a reporter (HRE-luc)^53^, the Topflash Wnt reporter^54^ or a RA signalling reporter plasmid (RARE-luc)^55^ and 0.02 μg of a renilla control vector using the Attractene Transfection Reagent (Qiagen). Luciferase and renilla activities were measured 48 h after transfection using the Dual-Luciferase Reporter Assay System (Promega).

### Quantitative reverse transcription polymerase chain reaction (qRT–PCR)

RNA was extracted from primary rib chondrocytes that isolated from neonatal mice using the Direct-zol RNA Mini-Prep Kit (Zymo Research). Quantitative reverse transcription polymerase chain reaction (qRT–PCR), RNA was reverse-transcribed using DyNAmo cDNA Synthesis Kit (Life Technologies) and real-time PCR was performed using the StepOnePlus Real-time PCR system (Life Technologies) and FirePol EvaGreen qPCR mix (Solis Biodyne). Values were normalized by Actb. Primer sequences of qRT–PCR primers are as follows: Actb, 5′-GCACTGTGTTGGCATAGAGG-3′ and 5′-GTTCCGATGCCCTGAGGCTCTT-3′; 18S ribosomal RNA, 5′-AAACGGCTACCACATCCAAG-3′ and 5′-CCTCCAATGGATCCTCGTTA-3′; Hif1a, 5′-TCAAGTCAGCAACGTGGAAG-3′ and 5′-TATCGAGGCTGTGTCGACTG-3′; Vegfa, 5′-GAGAGAGGCCGAAGTCCTTT-3′ and 5′-TTGGAACCGGCATCTTTATC-3′; Pdk1, 5′-GAAGCAGTTCCTGGACTTCG-3′ and 5′-CCAACTTTGCACCAGCTGTA-3′; Ndufa4l2, 5′-TGATTGGCTTCATCTGCTTG-3′ and 5′-ACTGGTCATTGGGACTCAGG-3′; Bnip3, 5′-GGGTTTTCCCCAAAGGAATA-3′ and 5′-GAATCCTCATCCTGCAAAGC-3′; Axin2, 5′-CTCCCCACCTTGAATGAAGA-3′ and 5′-ACTGGGTCGCTTCTCTTGAA-3′; Lef1, 5′-TCACTGTCAGGCGACACTTC-3′ and 5′-TGAGGCTTCACGTGCATTAG-3′; Tgfb1r, 5′-ACCTTCTGATCCATCGGTTG-3′ and 5′-CCTGTTGGCTGAGTTGTGAC; Tgfbr2, 5′-TCGGATGTGGAAATGGAAG-3′ and 5′-CTGGCCATGACATCACTGTT-3′; Wnt10a, 5′-CCACTCCTGGCCTGTCAC-3′ and 5′-AGCCAGCAGCAGTAGGAAGA-3′; Frz3, 5′-GGTGTCCCGTGGCCTGAAG-3′ and 5′-ACGTGCAGAAAGGAATAGCCAAG-3′; Frz6, 5′-CTTTTTGATGCGGAAAGGAG-3′ and 5′-TCTTACGAGGGGCAGAAGAA-3′; Lrp4, 5′-GAATGTGCTGAGGAGGGGTA-3′ and 5′-TTGGCAAACAGTAGCACAGG-3′; Lrp5, 5′-CTGTGGCTGTGCTTCACACT-3′ and 5′-CTTGTCCAGCGGGTCATAGT-3′; Cdkn2a 5′-GTACCCCGATTCAGGTGATG-3′ and 5′-GGAGAAGGTAGTGGGGTCCT-3′.

### Chromatin immunoprecipitation

ChIP was performed using the SimpleChIP Enzymatic Chromatin IP kit (#9003, Cell Signaling Technology), anti-H3K27me3 antibody (#9733, Cell Signaling Technology) and IgG. ChIP-ed DNA was quantified with qPCR. PCR primers used for qPCR are as follows: Wnt10a, 5′-CCACTCCTGGCCTGTCAC-3′ and 5′-AGCCAGCAGCAGTAGGAAGA-3′; Lef1, 5′-GCGAAAGGGAAGGAAAGAAG-3′ and 5′-GGATGCTGATTTCGGTGATT-3′; Lrp4, 5′-GAATGTGCTGAGGAGGGGTA-3′ and 5′-TTGGCAAACAGTAGCACAGG-3′; Lrp5, 5′-GCCGGACGACATGGAAAC-3′ and 5′-GGGACCAAGCTGCAGTACA-3′; Fzd3, 5′-CGGACTTTGCAAGAAGGACT-3′ and 5′-CCTGGCGTCCTAGGTGATAG-3′; Fzd6, 5′-GCCAGACTCCCCGAGTTAAT-3′ and 5′-ACACTTTCCGTTCTGGAAGC-3′; Tgfbr1, 5′-CCCCTCGAGCAGTTACAAAG-3′ and 5′-CCACCAACACGATGAGGAG-3′; Tgfbr2, 5′-CCGGGTAAAGTTGATGAGTGA-3′ and 5′-CCTTTACTCCTCGCCCTCTC-3′; Cdkn2a, 5′-ATCTGGAGCAGCATGGAGTC-3′ and 5′-GGGGTACGACCGAAAGAGTT-3′.

More information is available in the Supplementary Methods.

### Western blot analysis

Anti-Eed antibody (#09-774, rabbit polyclonal, 1:1,000) was purchased from Millipore. Anti-H3K27me3 antibody (GTX1121184, rabbit polyclonal, 1:500) was purchased from GeneTex. Anti-Actin antibody (I-19, rabbit polyclonal, 1:500) was purchased from Santa Cruz Biotechnology. Anti-Hif1a antibody (NB100-449, rabbit polyclonal, 1:500) was purchased from Novus Biologicals. Anti-Bnip3 antibody (ab10433, mouse monoclonal (ANa40), 2 μg ml^−1^) was purchased from Abcam. Anti-p-ERK1/2 antibody (#4370, rabbit monoclonal, 1:1,000), anti-p-MEK1 antibody (#9154, rabbit monoclonal, 1:1,000), anti-p-cRaf antibody (#9427, rabbit monoclonal, 1:1,000), anti-p-RSK antibody (#9335, rabbit monoclonal, 1:1,000) and anti-p-p38 antibody (#4511, rabbit monoclonal, 1:1,000), anti-total ERK antibody (#9102, rabbit polyclonal, 1:1,000), anti-p-Smad1/5/8antibody (#9511, rabbit polyclonal; 1:1,000), anti-Smad2 antibody (#5339, rabbit monoclonal, 1:1,000), anti-p-Smad2 antibody (#3104, rabbit polyclonal, 1:1,000), anti-TGF-β receptor II antibody (#3713, rabbit polyclonal, 1:1,000), anti-p-STAT1 antibody (#9171, rabbit polyclonal, 1:1,000) and anti-pSTAT3 antibody (#9145, rabbit monoclonal, 1:2,000) were purchased from Cell Signaling Technology. Western blot analysis was performed according to the standard procedure.

### Immunohistochemistry

Immunohistochemistry was performed on paraffin sections using Perkin Elmer Tyramide Signal Amplification Kit (# NEL700A001KT) according to the manufacturer's instruction. Anti-p-Smad2 (#3104, rabbit polyclonal, 1:200), anti-Tgfbr2 (#3713, rabbit polyclonal, 1:100) and Non-phospho (Active) β-Catenin (#D13A1, rabbit monoclonal, 1:500) were purchased from Cell Signalling Technology.

### Retrovirus generation and infection

Retroviruses expressing shRNA for *Tgrbr2*, and *Cdkn2*, were constructed using a modified pMSCV vector (Clontech)^56^. For shRNA constructs for *Tgfbr2* (Tgfbr2-sh1, -sh2 and -sh3) and Cdkn2a (Cdkn2a-sh1 and -sh2), the following sequences were synthesized and subcloned into pMSCV-EGFP:

Cdkn2a-sh1, 5′-GATGATGATGGGCAACGTTCACTCGAGTGAACGTTGCCCATCATCATC-3′; Cdkn2a-sh2, 5′-CTAGCGATGCTAGCGTGTCTACTCGAGTAGACA CGCTAGCATCGCTAG-3′; Tgfbr2-sh1, 5′-TGGCAGAAATTACAAGTGCATATTTCTCGAGAAATATGCACTTGTAATTTCTGCCA-3′; Tgfbr2-sh2, 5′-GTGTAAATACGAATAGCTATGTTCT CGAGAACATAGCTATTCGTATTTACACAC-3′ Tgfbr2-sh3, 5′-GTGGAGGAAGAACGACAAGAACATTCTCGAGAATGTTCTTGTCGTTCTTCCTCCAC-3′.

### Statistical analysis

Values are expressed as means±s.e.m. Statistical significance between two groups was determined by unpaired Student's *t*-test. The effect of the TGF-β inhibitor on occurrence of cell death in cKO was determined by *χ*^2^-test.

### Data availability

ChIP-seq and microarray data that support the findings of this study have been deposited in NCBI GEO with the primary accession codes GSE67132 and GSE66862, respectively.

## Additional information

**How to cite this article**: Mirzamohammadi, F. *et al*. Polycomb repressive complex 2 regulates skeletal growth by suppressing Wnt and TGF-β signalling. *Nat. Commun.* 7:12047 doi: 10.1038/ncomms12047 (2016).

## Supplementary Material

Supplementary Information Supplementary Figures 1-6, Supplementary Methods and Supplementary References.

Supplementary Data 1 Genes associated with H3K27me3 in chondrocytes. Annotated ChIP-seq data and comparison of ChiP-seq data with microarray analysis data (Supplementary Data2).

Supplementary Data 2 Gene expression profiling in Eed-deficient chondrocytes. Microarray analysis comparing control and Eed cKO chondrocytes.
