# Supplementary material for: Polycomb repressive complex 2 regulates skeletal growth by suppressing Wnt and TGF-β signalling
Source: Nat Commun. 2016 Jun 22;7:12047. doi: 10.1038/ncomms12047 (PMC4917962; doi:10.1038/ncomms12047)
Supplement: Supplementary Information — Supplementary Figures 1-6, Supplementary Methods and Supplementary References. [file ncomms12047-s1.pdf]

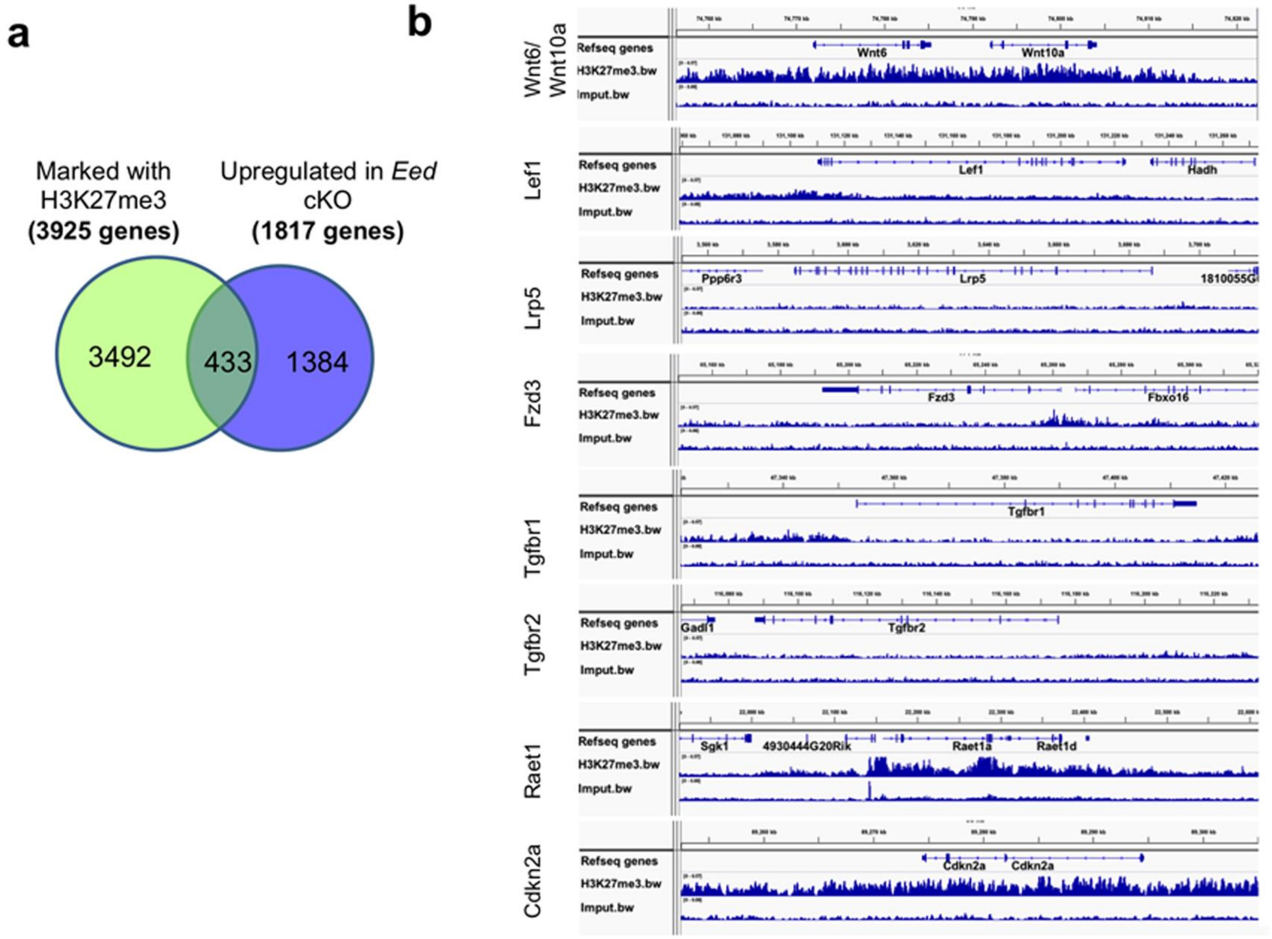

Supplementary Figure 1 (Related with Figure 4). **Molecular consequences of *Eed* deletion.** (a) ChIP analysis identifies 3925 genes that are associated with the H3K27me3 mark in chondrocytes (see Table S1, Experiment 2). Microarray analysis (see Table S2) identified 1817 upregulated genes that were upregulated more than 1.25 fold upon *Eed* deletion. Only 433 genes (12.4%) marked with H3K27me3 were upregulated in *Eed*-deficient chondrocytes. (b) Read mapping of indicated genomic loci.

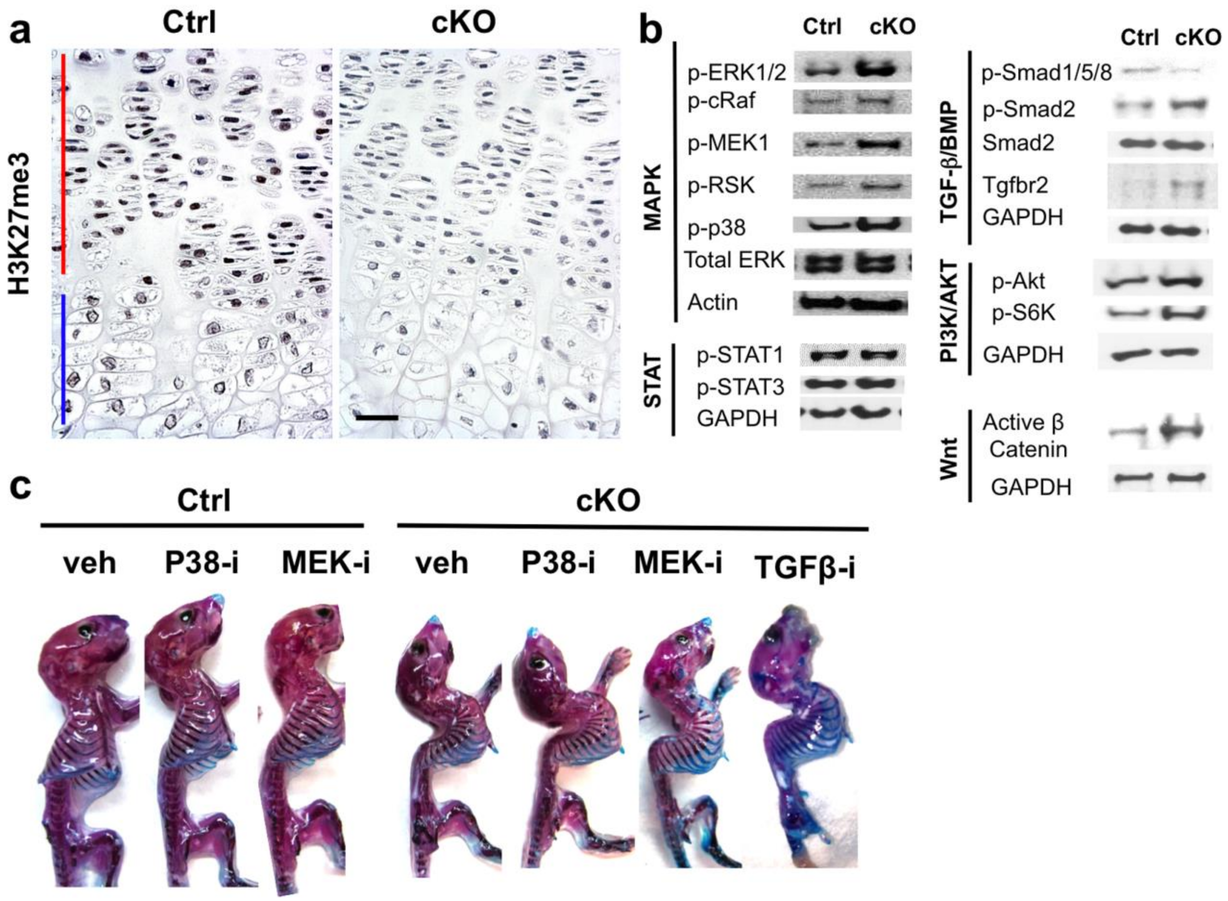

Supplementary Figure 2 (Related with Figures 1,4,5 and 6). (a) Immunostaining for H3K27me3 on tibial growth plate sections of wild type (Ctrl) and *Eed* cKO (cKO) mice. The red bar indicates the proliferating chondrocyte zone and the blue bar indicates the hypertrophic chondrocyte zone. Scale bar, 20 µm. (b) Immunoblot analysis using protein lysates of primary rib chondrocytes cultured overnight shows aberrant activation of multiple signaling pathways in *Eed* cKO chondrocytes. (c) TGF-β inhibitors (TGFβ-i; LY364947, 1 ug/g BW), a p38 inhibitor (p38-i; SB203580, 25 ug/g BW), or a MEK inhibitor (MEK-i; U0126, 5 ug/g BW) was intraperitoneally injected daily to pregnant and nursing mothers from E 14.5 through P5.5, and then directly to the pups until P9.5. Mice were sacrificed at P9.5 for evaluation. The dosage of U0126 was determined according to previous reports in which U0126 injection successfully rescued skeletal abnormalities caused by ERK overactivation[1,2]. Likewise, p38 inhibitor injection protocol was determined according to the study that demonstrated that SB203580 injection rescued skin and skeletal phenotypes caused by a Fgfr2 overactivation mutation in mice [3]. The dosage of the TGF-β receptor inhibitor, Ly364947, was determined according to a previous study [4].

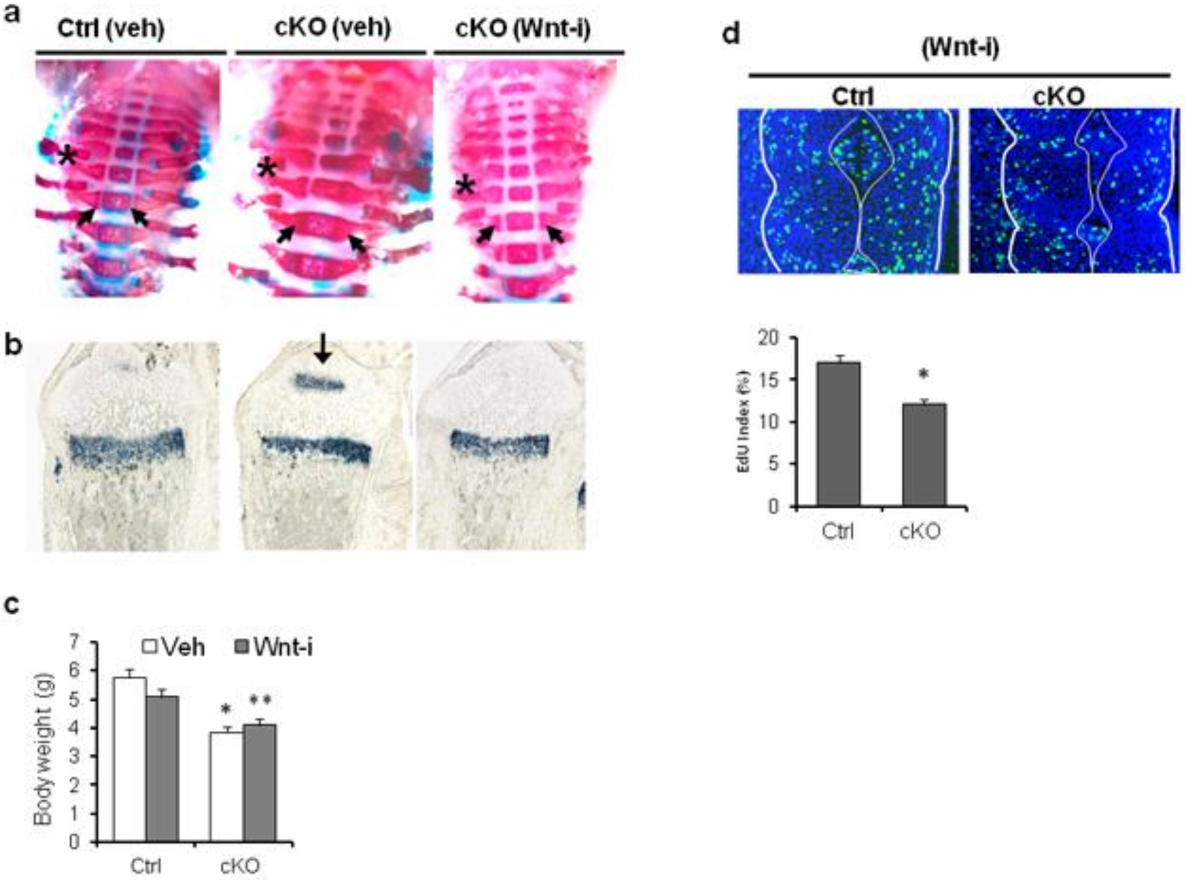

Supplementary Figure 3 (Related with Figure 6). **Wnt inhibition ameliorates kyphosis of *Eed* cKO mice**. (a) Frontal views of cervical spine of P7.5 –old mice with indicated genotypes treated with vehicle (Veh) or C59 Wnt inhibitor (Wnt-i). Asterisks indicate the first rib. Arrows show cartilage between the vertebral body and transverse processes, which is prematurely replaced by mineralized bone in vehicle-treated cKO mice. Wnt inhibitor treatment restores this cartilage. (b) Wnt inhibitor treatment rescues premature hypertrophic differentiation in the prospective secondary ossification center of the *Eed*-deficient tibial epiphysis (arrow). (c) Wnt inhibitor treatment was not able to significantly improve animal growth. $n = 6$ each group. * $p < 0.05$ vs Ctrl treated with vehicle, ** $p < 0.05$ vs Ctrl treated with vehicle or C59 (Wnt-i). (d) Chondrocyte proliferation in the spinal primordium assessed by EdU incorporation at E15.5 after injection of C59 Wnt inhibitor for two days. Wnt inhibition did not rescue the proliferation defect of vertebral chondrocytes of *Eed* cKO embryos. Thick white lines indicate the contour of the spinal primordium at the level of C4-C5. Thin lines indicate the notochord. $n = 3$; * $p < 0.001$ vs Ctrl.

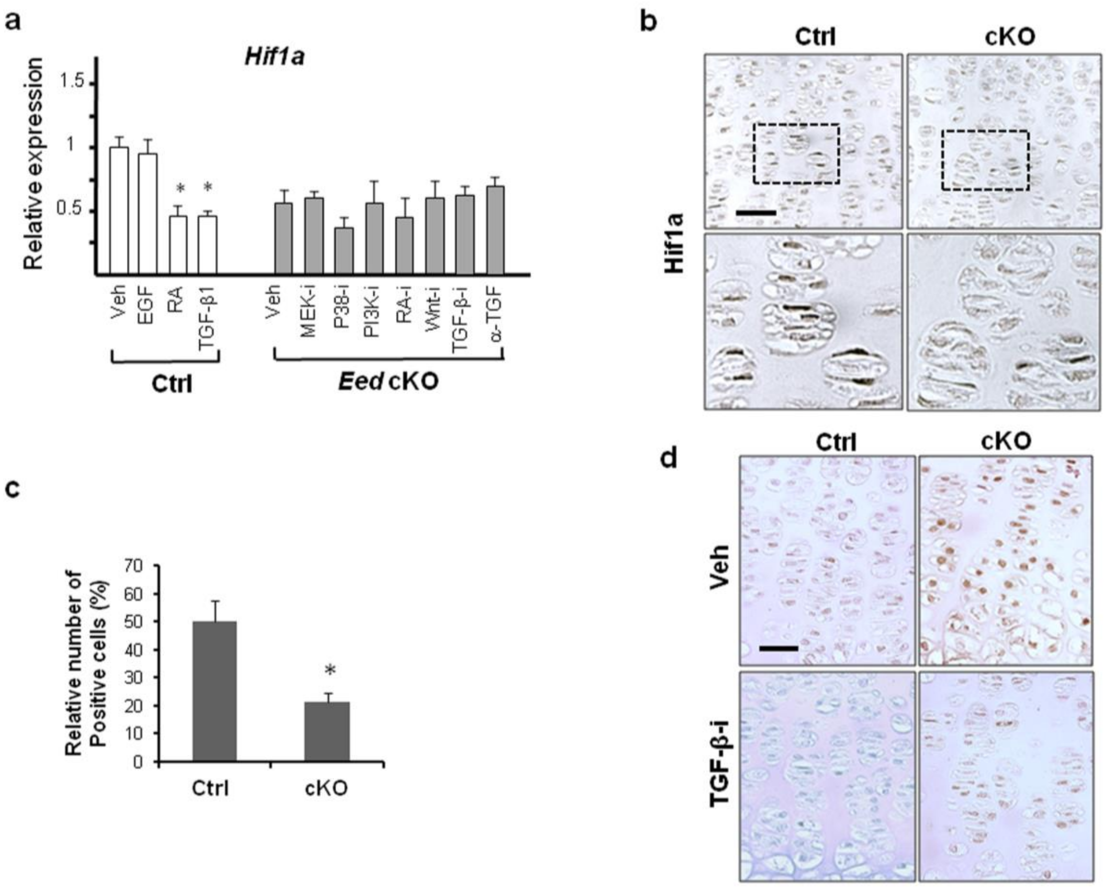

Supplementary Figure 4 (Related with Figures 3 and 5). (a) Effects of signaling manipulation on *Hif1a* expression. Although retinoic acid (RA) or TGF-β1 treatment reduces *Hif1a* expression in control (Ctrl) cells, inhibition of indicated signaling pathways fails to restore *Hif1a* expression in primary rib chondrocytes. *Hif1a* expression was determined by qRT-PCR upon treatment with indicated ligands or inhibitors for 24 hrs. Cells were treated with TGF-β1 (50 ng/ml), RA (12.5 uM), EGF (5 ng/ml), MEK inhibitor (MEK-i; U0126, 10 nmol/ml), p38 inhibitor (p38-i; SB203580, 5 mM), PI3K inhibitor (PI3K-i; LY294002, 50 mM), RA inhibitor (RA-i; AGN-193109 2uM and BMS-493 10 uM), Wnt inhibitor (Wnt-i; C59 100 nM), Tgfbr2 inhibitor (TGFβ-i; Ly364947 0.3 uM), and TGF-β lignad neutralizing antibody (αTGF; 10 ug/mL). $n = 4$; * $p < 0.05$ vs vehicle treated sample of each genotype (veh). (b) Immunostaining for Hif1a protein in Ctrl and cKO tibial growth plate sections of P7.5-old mice. Magnified views of boxed regions are shown in lower panels. (c) Quantification of cells with strong nuclear Hif1a staining in Ctrl and cKO (n=3). * $p < 0.05$. Staining is assessed by setting a “threshold” using the ImageJ software. The number of stained cells above a given intensity threshold is considered positive and the proportion of the positive cells to the total number of cells in each section presented as percentage of positive cells. Samples preparation, processing, sectioning, staining and measurement settings are all identical in both Ctrl and cKO groups. *p<0.001 vs. Ctrl. (d) Tibial growth plate sections from Ctrl and cKO mice treated with vehicle (Veh) or TGF-β receptor inhibitor (TGFβ-i; Ly364947 1ug/g BW, ip) were stained for p-Smad2 (n=3). Mice were sacrificed two hours after TGFβ-i treatment.

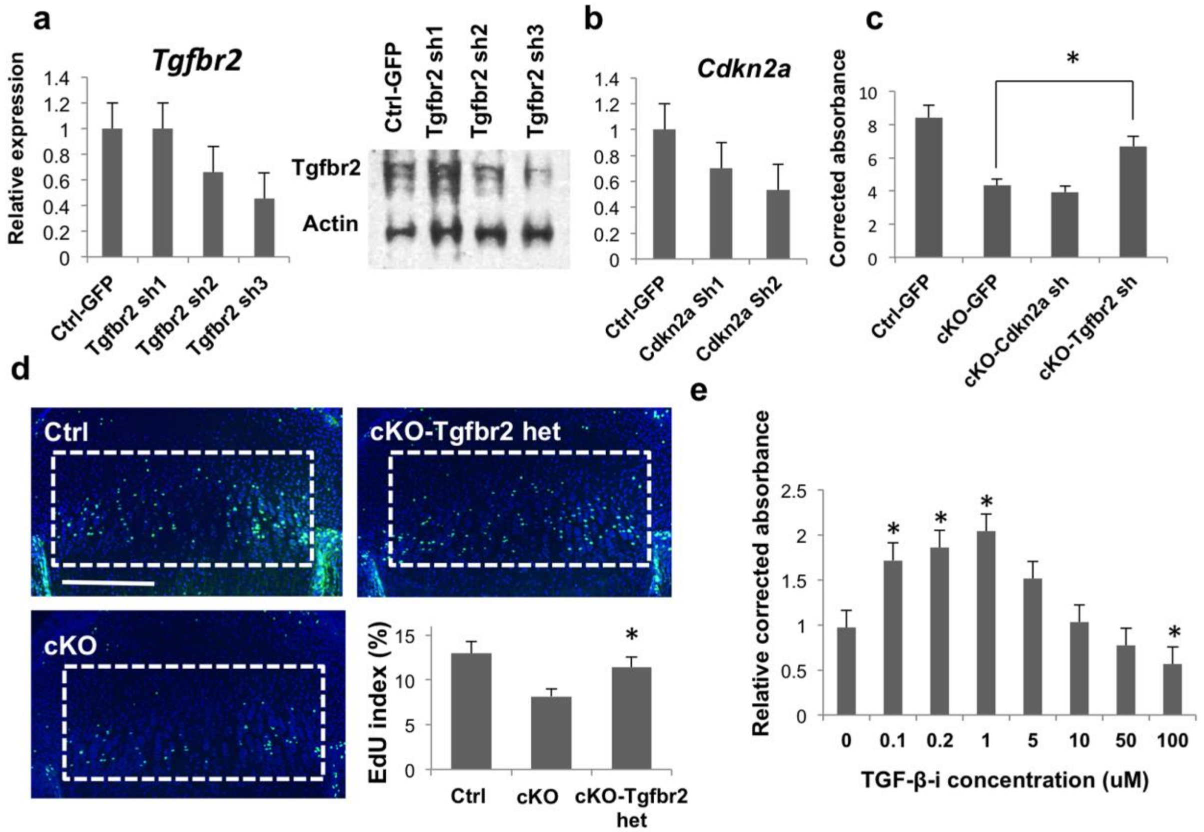

Supplementary Figure 5 (Related with Figures 5). **Upregulation of *Tgfbr2* is responsible for the proliferation defect of Eed deficient chondrocytes**. (a) Three different *Tgfbr2* shRNA and GFP-shRNA retroviral constructs were tested for knockdown efficiency. Two days after viral infection to primary rib chondrocytes, Tgfbr2 mRNA (left panel) and protein (right panel) levels were assessed. With the condition in which the maximal infection efficiency (60%, assessed by GFP), 40-60 % reduction in *Tgfbr2* expression was achieved by *Tgfbr2* sh2 and sh3 viruses.
(b) Two different *Cdkn2a* shRNA constructs were similarly generated. With the infection efficiency of 60%, 50% reduction in *Cdkn2a* expression level achieved in primary rib chondrocytes. (c) MTS cell proliferation assay on control (Ctrl) and Eed cKO (cKO) chondrocytes infected with *Tgfbr2* sh2 (Tgfbr2 sh), *Cdkn2a* sh2 (Cdkn2a sh), or GFP retroviruses. *Cdkn2a* knockdown had little effect in chondrocyte proliferation while *Tgfbr2* knockdown showed a significant increase in chondrocyte proliferation *in vitro*. Cell proliferation assay was performed at day 0 and day 4 after infection. Values were normalized by those at day 0. (d) *Tgfbr2* heterozygosity (cKO-Tgfbr2 het) significantly improved animal growth and growth plate chondrocyte proliferation of Eed cKO mice. EdU staining of the tibial growth plate of mice with indicated genotypes. n ≥ 5 in each group . $p < 0.05$ cKO-Tgfbr2 het vs. cKO. Scale bar, 200 μm. (e) The effect of the TGF-β inhibitor (TGFβ-i; Ly364947) at indicated concentrations on chondrocytes proliferation assessed by MTS assay *in vitro*. TGFβ-i treatment increases chondrocyte proliferation at lower concentrations, whereas it decreases proliferation at higher concentrations (> 50uM). n = 6, $*p < 0.05$.

Fig. 1f

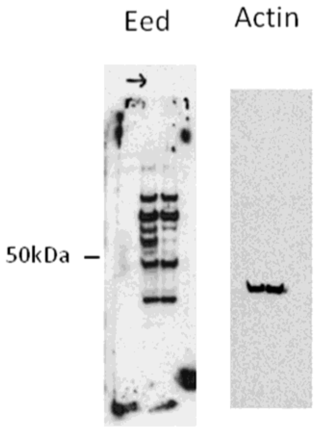

Fig. 1h

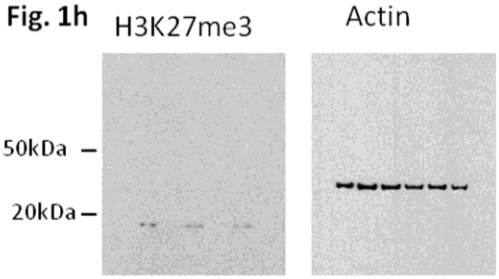

Fig. 3c

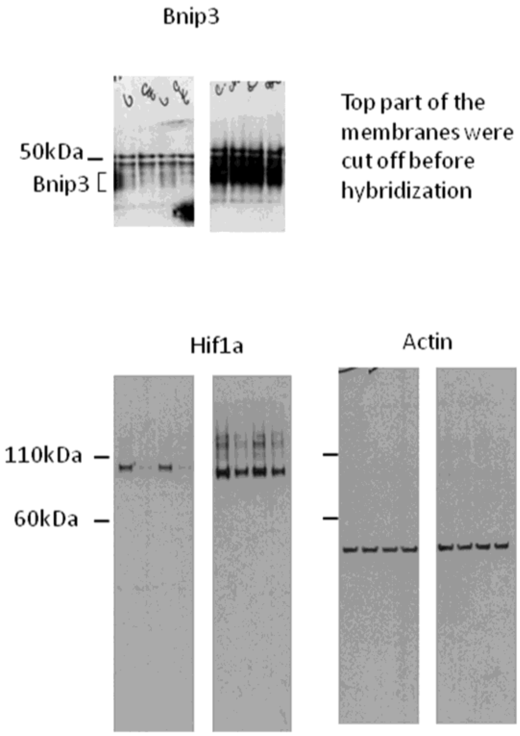

Fig. 4a

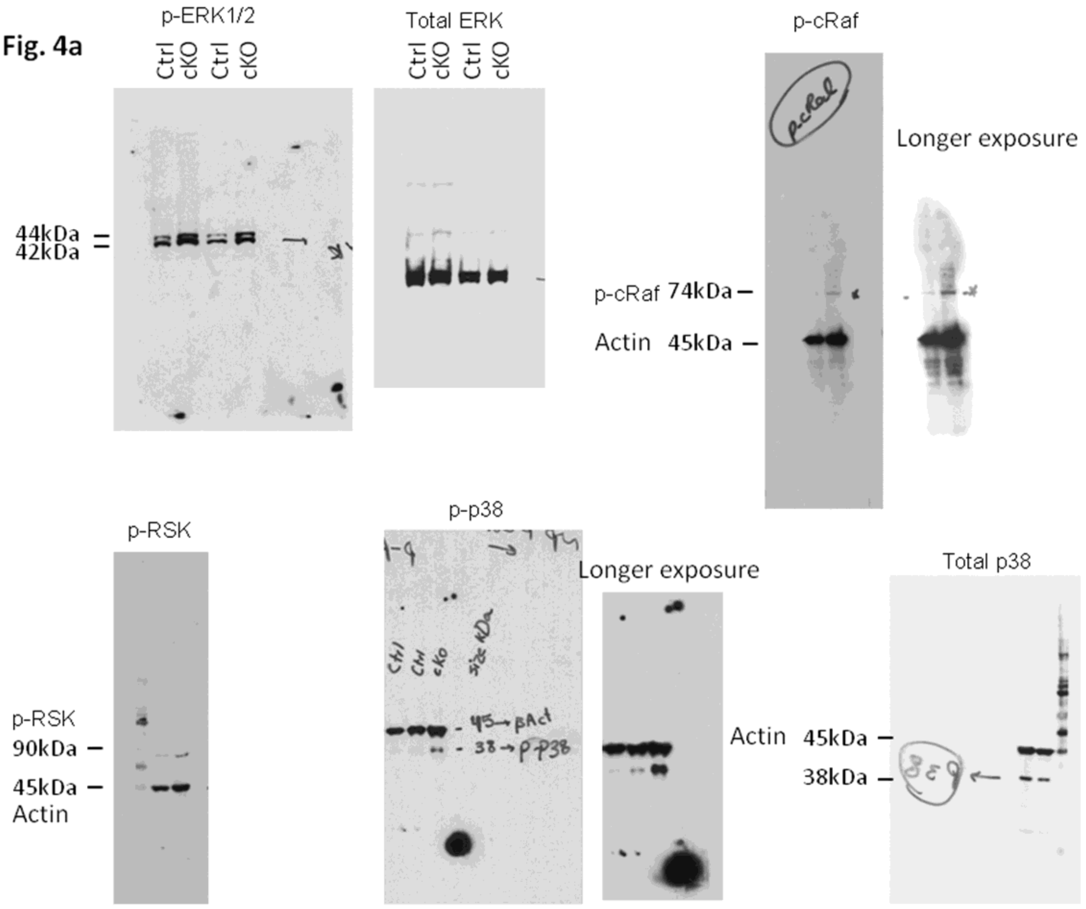

p-ERK1/2
Ctrl cKO Ctrl cKO
44kDa
42kDa
Total ERK
Ctrl cKO Ctrl cKO
p-cRaf
Longer exposure
p-cRaf 74kDa
Actin 45kDa
p-RSK
p-RSK
90kDa
45kDa
Actin
p-p38
Longer exposure
Actin
Total p38
45kDa
38kDa

Fig. 4a

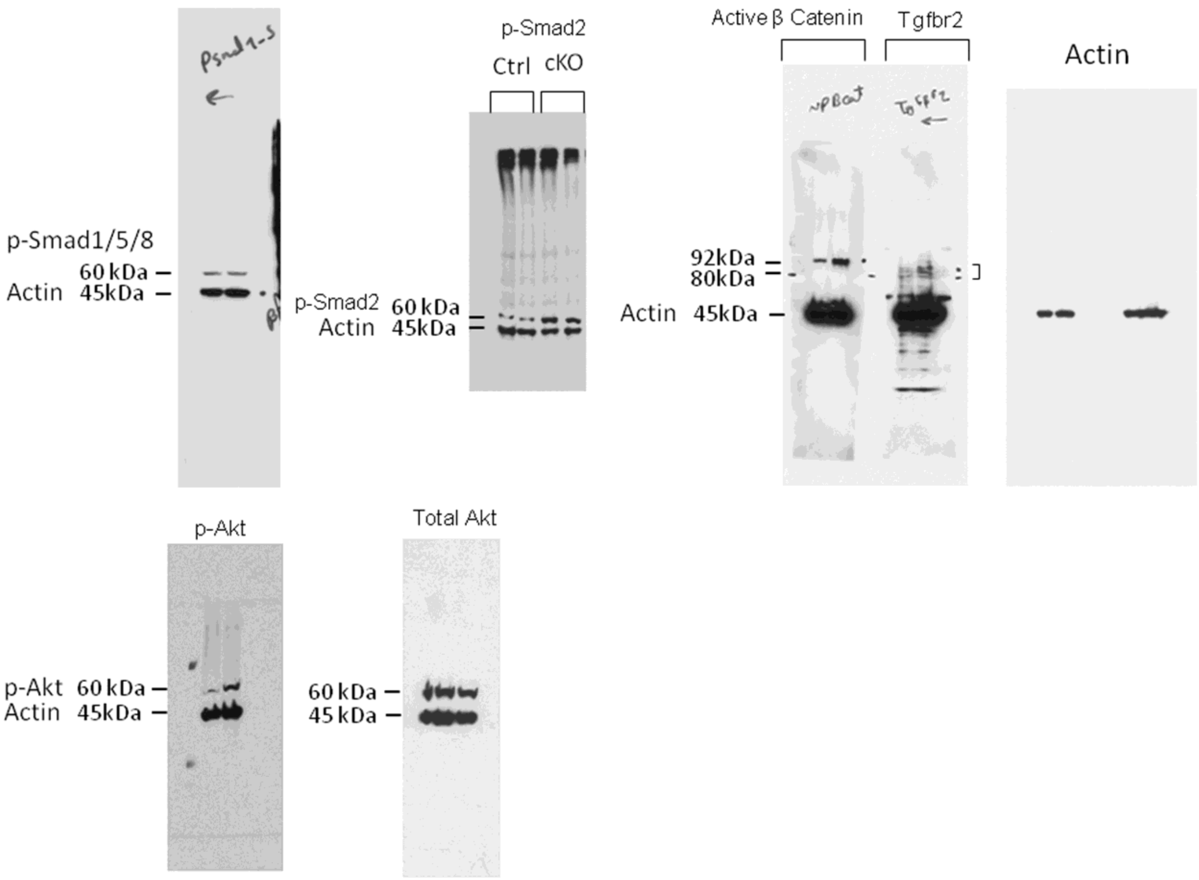

Fig. 5d

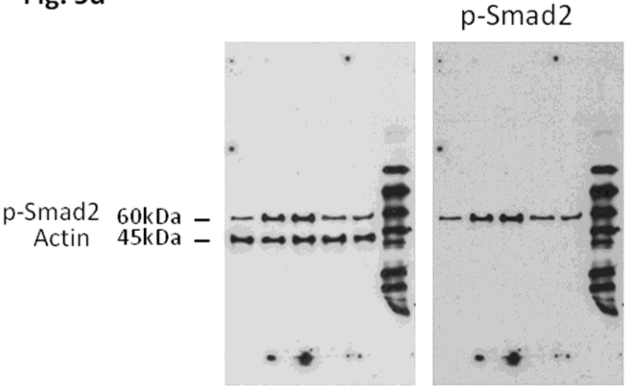

Fig. 5f

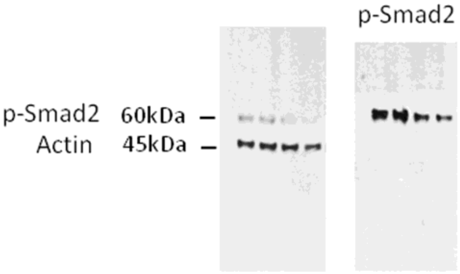

Supplementary Figure 6. The uncropped blot result images. Related to the Figure 1, 3, 4 and 5.

Supplementary Methods

Chromatin immunoprecipitation (ChIP) and sequencing (ChIP-seq)

Primary rib chondrocytes isolated from P2.5 CD-1 mice were subjected to ChIP-seq analysis using the SimpleChIP Enzymatic Chromatin IP kit (#9003, Cell Signaling Technology) and anti-H3K27me3 antibody (#9733, Cell Signaling Technology. ChIP'ed DNA and input DNA were sequenced by the Illumina Hi-Seq 2500 sequencer at Otogenetics (GA). Reads were mapped to the mouse genome (Build 38/mm10) using the Bowtie2. MACS2 was used for peak calling with the q value smaller than 0.05. Peak regions were annotated using PAVIS [5]. A called peak was associated with a gene when the peak was located within the region of 5kb-upstream and 1kb-downstream 1kb of a gene.

Microarray Analysis and quantitative reverse transcription polymerase chain reaction (qRT-PCR) analysis

RNA was extracted from primary rib chondrocytes from newborn mice. Chondrocytes were cultured in DMEM containing 10% fetal calf serum over night. RNA was extracted using Direct-zol RNA mini-prep kit (Zymo Research). Gene expression profiling was performed using the Affymetrix Mouse Gene 1.0 ST.

Immunostaining. Immunohistochemistry was performed on paraffin sections using Perkin Elmer Tyramide signal amplification kit (# NEL700A001KT) according to manufacture's instruction. Anti-Hif1a antibody (# NB100-449, Novus; 1:000) and anti-H3K27me3 antibody (# GTX121184, Gen Tex; 1:1000) were purchased.

Retrovirus generation. Retroviruses expressing shRNA for *Tgrbr2*, *Cdkn2a,* and e*GFP* were constructed using a modified pMSCV vector (Clontech) [6]. For shRNA constructs for *Tgfbr2* (Tgfbr2-sh1, -sh2, and -sh3) and Cdkn2a (Cdkn2a-sh1, and -sh2), the following sequences were synthesized and subcloned into pMSCV-EGFP:
Cdkn2a-sh1**,**5'-GATGATGATGGGCAACGTTCACTCGAGTGAACGTTGCCCATCATCATC -3';
Cdkn2a-sh2, 5'- CTAGCGATGCTAGCGTGTCTACTCGAGTAGACACGCTAGCATCGCTAG-3';
Tgfbr2-sh1, 5'-
TGGCAGAAATTACAAGTGCATATTTCTCGAGAAATATGCACTTGTAATTTCTGCCA-3';
Tgfbr2-sh2, 5'-
GTGTAAATACGAATAGCTATGTTCTCGAGAACATAGCTATTCGTATTTACACAC-3' Tgfbr2-sh3, 5'-GTGGAGGAAGAACGACAAGAACATTCTCGAGAATGTTCTTGTCGTTCTTCCTCCAC-3'

Supplementary References

1. Shukla, V., Coumoul, X., Wang, R.H., Kim, H.S. & Deng, C.X. RNA interference and inhibition of MEK-ERK signaling prevent abnormal skeletal phenotypes in a mouse model of craniosynostosis. *Nat Genet* **39**, 1145-1150 (2007).
2. Nakamura, T., Gulick, J., Pratt, R. & Robbins, J. Noonan syndrome is associated with enhanced pERK activity, the repression of which can prevent craniofacial malformations. *Proc Natl Acad Sci U S A* **106**, 15436-15441 (2009).
3. Wang, Y.*, et al.* p38 Inhibition ameliorates skin and skull abnormalities in Fgfr2 Beare-Stevenson mice. *J Clin Invest* **122**, 2153-2164 (2012).
4. Oka, M.*, et al.* Inhibition of endogenous TGF-beta signaling enhances lymphangiogenesis. *Blood* **111**, 4571-4579 (2008).
5. Huang, W., Loganantharaj, R., Schroeder, B., Fargo, D. & Li, L. PAVIS: a tool for Peak Annotation and Visualization. *Bioinformatics* **29**, 3097-3099 (2013).
6. Papaioannou, G., Inloes, J.B., Nakamura, Y., Paltrinieri, E. & Kobayashi, T. let-7 and miR-140 microRNAs coordinately regulate skeletal development. *Proc Natl Acad Sci U S A* **110**, E3291-3300 (2013).
